# Acute Local Spontaneous and Profuse Gingival Hemorrhage during Neoadjuvant Treatment with Paclitaxel and Trastuzumab

**DOI:** 10.3390/dj4030022

**Published:** 2016-06-24

**Authors:** Nima D. Sarmast, Maria J. Gutierrez Quevedo, Howard H. Wang, Estatio R. Gutierrez Herrera

**Affiliations:** 1 Department of Periodontics and Dental Hygiene, The University of Texas School of Dentistry at Houston, Houston, TX 77054, USA; 2 Private Dental Practice, Houston, TX 77064, USA; mariajgutierrez29@gmail.com; 3 Private Dental Practice, New York, NY 10002, USA; haowangdds@gmail.com; 4 Centro Medico Loira, Caracas 1020, Venezuela; egutierrezve@hotmail.com

**Keywords:** gingival diseases, periodontal diseases, gingival hemorrhage, breast cancer, chemotherapy

## Abstract

This case report describes a 33-year-old female currently undergoing breast cancer treatment following the AC-T-T (doxorubicin hydrochloride (Adriamycin) and cyclophosphamide followed by paclitaxel (Taxol) and trastuzumab (Herceptin)) treatment regimen. Her chief complaint at the time of the emergency visit at the dental office was that she had an episode of profuse spontaneous bleeding located at the palatal gingiva in the maxilla between the left central and lateral incisor. To our knowledge, this is a novel finding related to the medications she is utilizing and should be further investigated.

## 1. Introduction

Periodontal diseases and conditions, in part, may have different etiological origins and manifestations. The more common types are plaque-induced gingivitis and chronic periodontitis, both caused by interactions between the bacterial biofilm on teeth and the host immune response [1,2]. Gingival diseases modified by systemic factors or medications and gingival manifestations of systemic conditions are forms of periodontal conditions that affect the periodontium, the specialized tissues that surround and support the dentition within the jaws [1]. Gingival disease can be modified by systemic factors including changes in hormone levels such as during puberty, menses or pregnancy which could result in the formation of pyogenic granulomas [3,4,5]. Another example is leukemia-associated gingivitis, where rapidly forming gingival hyperplasia could be the first sign of this disease, which manifests intra-orally [6,7]. Drug-induced gingival enlargement typically involves medications such as anticonvulsants, the immunosuppressant cyclosporine, and calcium channel blockers. Systemic mucocutaneous disorders such as lichen planus, pemphigus vulgaris, pemphigoid, erythema multiforme, lupus erythematosus and others may also present with various forms of pathologic reactions in the oral and perioral region [5]. In addition, adverse drug reactions can also exhibit debilitating oral manifestations such as increased bleeding, painful ulcerations and swelling, among others. As more links between systemic and oral conditions are being unveiled, there is a need for interdisciplinary collaboration in order to prevent and treat diseases and to restore healthy conditions in patients with concomitant conditions [5,6,7]. This may be especially evident in cases where multiple treatment modalities and medications are utilized such as the care of cancer patients. This case report describes an episode of acute gingival hemorrhage in an otherwise healthy 33-year-old female diagnosed with human epidermal growth factor receptor 2 (HER2)-positive invasive ductal carcinoma and undergoing 12 weeks of neoadjuvant therapy with weekly paclitaxel and triweekly trastuzumab, a monoclonal antibody and HER2/neu receptor inhibitor.

## 2. Case Presentation

**Figure A1:**
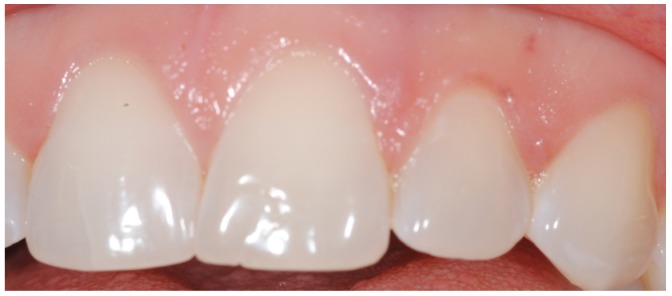
The buccal aspect of the region of interest after hemostasis was achieved.

**Figure A2:**
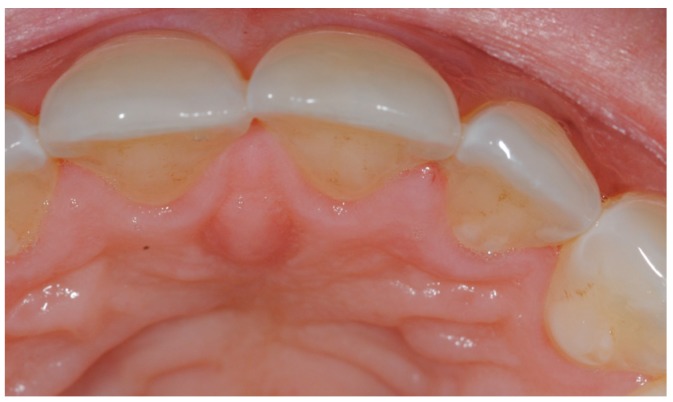
Palatal aspect of the region of interest after hemostasis was achieved.

**Figure A3:**
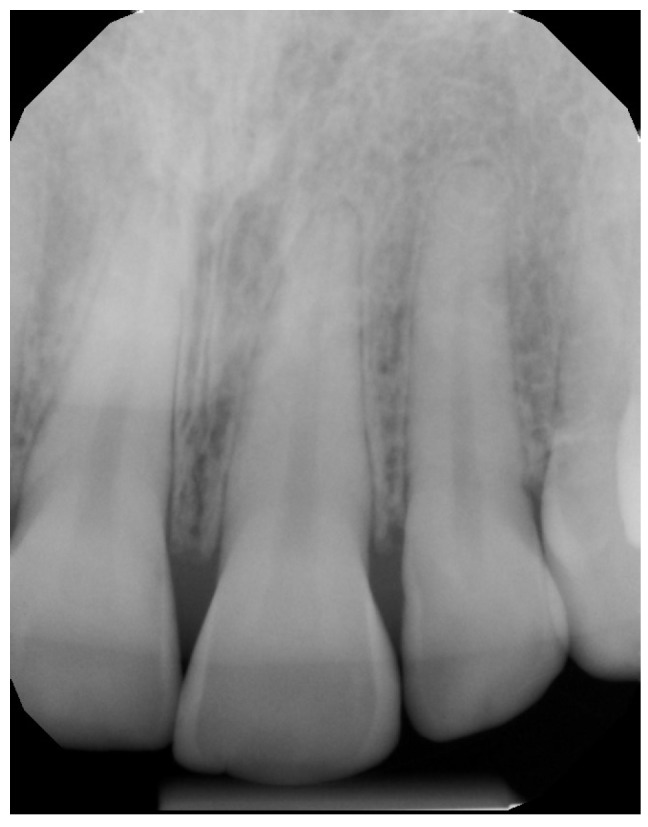
Periapical radiograph of the region of interest.

This case report describes a 33-year-old female currently undergoing breast cancer treatment following the AC-T-T (doxorubicin hydrochloride (Adriamycin) and cyclophosphamide followed by paclitaxel (Taxol) and trastuzumab (Herceptin)) treatment regimen at the University of Texas MD Anderson Cancer Center in Houston, Texas, USA. She reported an episode with profuse spontaneous bleeding located at the palatal gingiva in the maxilla between the left central and lateral incisor. The patient had no other known medical or oral conditions except for breast cancer, which was treated with 12 weekly infusions of paclitaxel and trastuzumab (T-T) every three weeks prior to her scheduled mastectomy. Her last infusion of T-T was six days prior to the incidence of the intraoral bleeding. The patient reported spontaneous bleeding from her mouth as she was getting dressed in the morning, which occurred before brushing her teeth or receiving any oral stimulus that may cause gingival bleeding, and became very concerned as the bleeding was profuse and difficult to control. A complete clinical oral examination was performed by a periodontist within an hour of the bleeding incident. The probing depths of teeth adjacent to the bleeding site were within normal limits, ranging from 2 to 3 mm, and no visible plaque was detected. A periapical intraoral radiograph from the anterior maxillary area revealed no signs of bone loss or other signs of pathology (Appendix A, Figure A1, Figure A2 and Figure A3). The patient has no previous history of periodontal disease, no trauma to the area and no para-functional habits, excellent oral hygiene, and there was no evidence of increased pocket probing depths or gingival inflammation or infection noted. There were neither reports of pain nor discomfort. The patient reported previous episodes of minor epistaxis that started after initiating treatment with the PT regimen, but no prior history of gingival bleeding. The patient was also not menstruating at this time. The patient started to bleed profusely again upon examination and gentle probing of the gingiva by the periodontist. The bleeding source was located at an area in the palatal gingiva between the left central maxillary incisor and the left lateral maxillary incisor. Applying continuous compression to the area with sterile gauze for over 20 min controlled the bleeding that was very copious from this source. The patient was monitored for 1 h prior to dismissal and was instructed to gently rinse the area with chlorhexidine 0.12% (twice daily for one week) alongside weekly monitoring. A complete blood count was ordered by her oncologist and revealed findings within normal levels with normal cell counts. The platelet count was 366 K/uL and the prothrombin time and activated partial thrombplastin time were all within range. There were no further reported episodes of intraoral bleeding or signs of gingival disease at subsequent follow-up visits at one, two and four weeks. The patient was advised to return for immediate follow-up if additional episodes of intra-oral bleeding occur.

## 3. Discussion

Acute spontaneous and profuse hemorrhage from the gingiva is a rare and often dramatic clinical occurrence that is disturbing for the patient. While likely not life threatening, it may be difficult for the patient to prevent and control it and it may cause an already stressful cancer life situation to further escalate. To our knowledge there are currently no cases in the literature reporting adverse reactions related to the oral cavity associated with trastuzumab except for reports of mucosal inflammation of the body as a whole recorded among the extensive potential adverse effects of this essential drug [8]. Paclitaxel, on the other hand, has unusual bruising or bleeding listed among its potential adverse effect as well as the possibility of ulcers, sores, or white spots in the mouth [9]. The patient’s episode of spontaneous and unusual bleeding could not be explained by thrombocytopenia or the presence of gingival inflammation and is thus attributed to other pharmacodynamic effects of paclitaxel not covered in this case report [10]. While sudden nosebleeds in conjunction with chemotherapy, especially paclitaxel, have been described in the literature, to our knowledge few references to intraoral bleeding exist for this drug. This may be attributed to being a rarity or the underreporting of cases. It may be prudent to convey to patients that possible side effects of paclitaxel may manifest as intraoral hemorrhage, in an effort to reduce the stress and anxiety often associated with unexplained bleeding, and to recommend involving dental professionals in the management of such cases to rule out other causes. This is, to our knowledge, the first case reporting acute spontaneous and profuse gingival hemorrhage and its clinical management in conjunction with the T-T treatment regimen in a breast cancer patient.
